# Neuroendocrine Cancer of Rectum Metastasizing to Ovary

**DOI:** 10.1155/2016/7149821

**Published:** 2016-05-18

**Authors:** Sapna Vinit Amin, Aswathy Kumaran, Sunanda Bharatnur, Akhila Vasudeva, Kartik Udupa, Dinesh Bangalore Venkateshiah, Shaila T. Bhat

**Affiliations:** ^1^Department of OBG, Kasturba Medical College, Manipal University, Manipal 576104, India; ^2^IVF and Reproductive Biology Centre, Maulana Azad Medical College, New Delhi 110002, India; ^3^Department of Medical Oncology, Kasturba Medical College, Manipal University, Manipal 576104, India; ^4^Department of Surgery, Kasturba Medical College, Manipal University, Manipal 576104, India; ^5^Department of Pathology, Melaka Manipal Medical College, Manipal University, Manipal 576104, India

## Abstract

Neuroendocrine carcinomas (NECs) are rare malignancies that originate from the hormone-producing cells of the body's neuroendocrine system. Rectal high grade NEC (HG-NEC) constituting less than 1% of colorectal cancers can cause large ovarian metastasis that may be the initial presenting complaint. Ovarian Krukenberg tumor from a primary rectal HG-NEC is a very unusual and exceedingly uncommon differential diagnosis for secondary ovarian malignancy. This case report describes one such extremely rare case of a woman who had presented to the gynecology department with features suggestive of ovarian malignancy and was ultimately diagnosed to have Krukenberg tumor originating from neuroendocrine cancer of rectum. We felt this is a good opportunity to spread more light on neuroendocrine neoplasms that are very rare in gynecological practice.

## 1. Introduction

Neuroendocrine neoplasms (NENs) are rare and fascinating neoplasms that begin in the hormone-producing cells of the body's neuroendocrine system, which is made up of cells that share the features of traditional endocrine cells (or hormone-producing cells) and nerve cells. They are multifaceted diseases that can primarily localize in many organs like the gastrointestinal tract, lungs, or brain and have various presentations. The most common neuroendocrine neoplasms are gastroenteropancreatic NENs (GEP-NENs). Based on the cell differentiation and immunohistochemistry, the GEP-NENs are differentiated into the slow growing well-differentiated neuroendocrine tumors, or WD-NETs, and the aggressive high grade/poorly differentiated neuroendocrine carcinomas, HG-NECs, that easily metastasize. The HG-NECs comprise less than one percent of the elusive neuroendocrine neoplasms (NENs).

This case report describes a woman with secondary ovarian malignancy that was neuroendocrine in origin, the primary being rectal HG-NEC. The GEP-neuroendocrine cancers metastasizing to ovaries make it extremely uncommon differential diagnosis for secondary ovarian neoplasms and hence the novelty of this case report.

Unfortunately, they have a very poor prognosis. Often only palliative chemotherapy is the therapeutic option, with 5-year survival being 12%.

## 2. Case Report

**Figure 1:**
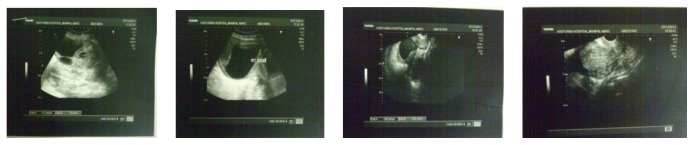
Ultrasonography.

A 42-year-old parous woman presented with chief complaints of abdominal distension and abdominal pain over a period of the last six months to the gynecology department of our tertiary level teaching hospital in 2013. General examination yielded nothing significant. On abdominal examination, there was a solid firm mass corresponding to 24 weeks' uterine size with ill-defined borders. Bimanual pelvic examination divulged bilateral forniceal fullness and rectal examination revealed a growth on the left lateral rectal wall. Abdominopelvic ultrasound showed bilateral complex adnexal masses (Figure 1) as well as moderate ascites. Tumor markers showed an elevated CA 125 of 195.3 U/mL. Liver and renal function tests were normal. Thus, we made a provisional diagnosis of ovarian malignancy.

**Figure 2:**
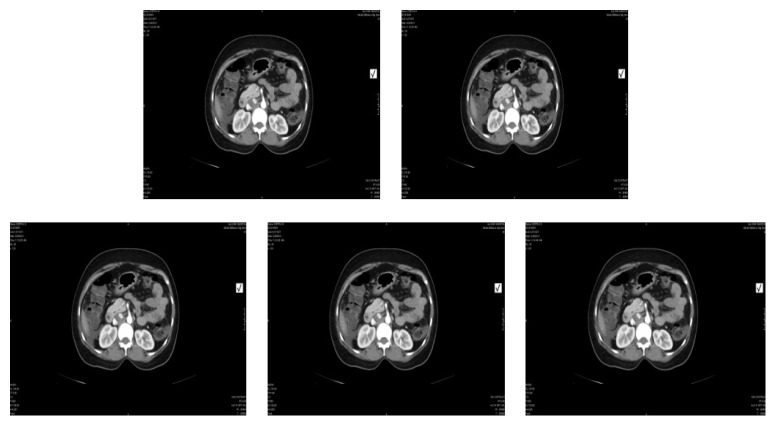
CT scan (abdomen and pelvis).

CT scan done showed two heterogeneously enhancing solid cystic lesions arising from the adnexae on either side measuring 12 × 9 cm on left and 9.7 × 8.5 cm on right, eccentric asymmetric wall thickening along the right posterolateral wall in the anal canal and rectum for a length of approximately 3-4 cm of maximal thickness 1.3 cm, and multiple hepatic metastatic lesions noted (Figure 2). This raised suspicion of primary rectal malignancy with bilateral metastatic ovarian tumor (Krukenberg) and liver metastasis.

**Figure 3:**
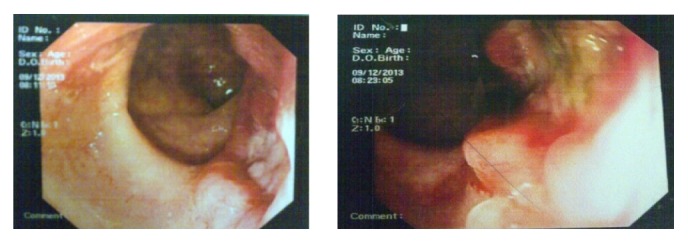
Sigmoidoscopy.

The ascitic fluid was positive for malignant cytology. Sigmoidoscopy (Figure 3) showed ulceroproliferative growth 1 cm from anal verge from which multiple biopsies were taken. The endocrine profile was normal and not suggestive of abnormal hormone production.

**Figure 4:**
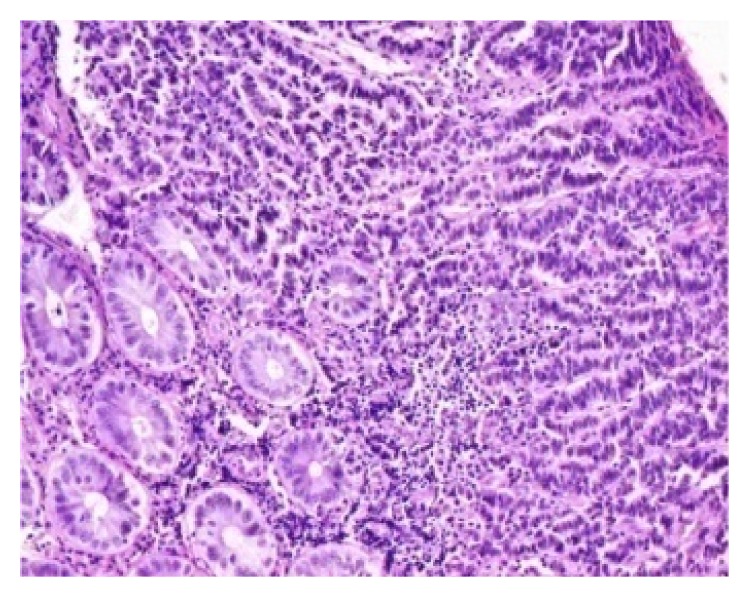
Photomicrograph showing neuroendocrine carcinoma involving the rectum.

**Figure 5:**
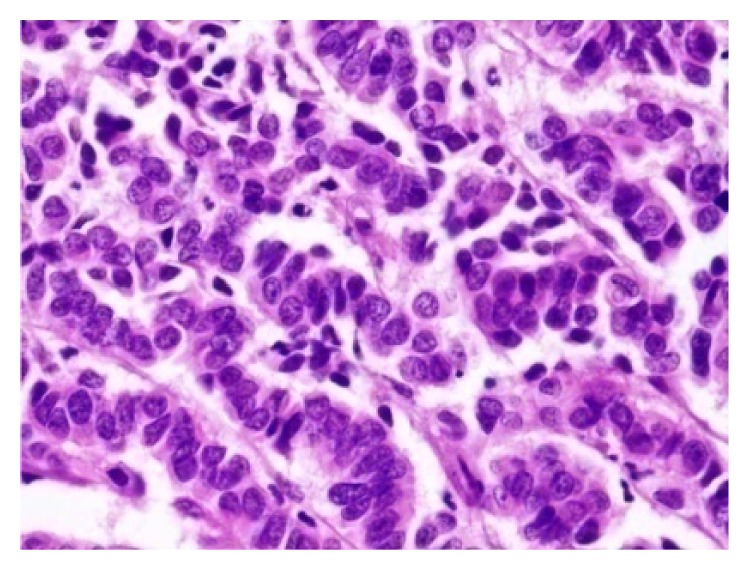
Photomicrograph showing tumor composed of cells with round to oval nuclei and stippled chromatin.

**Figure 6:**
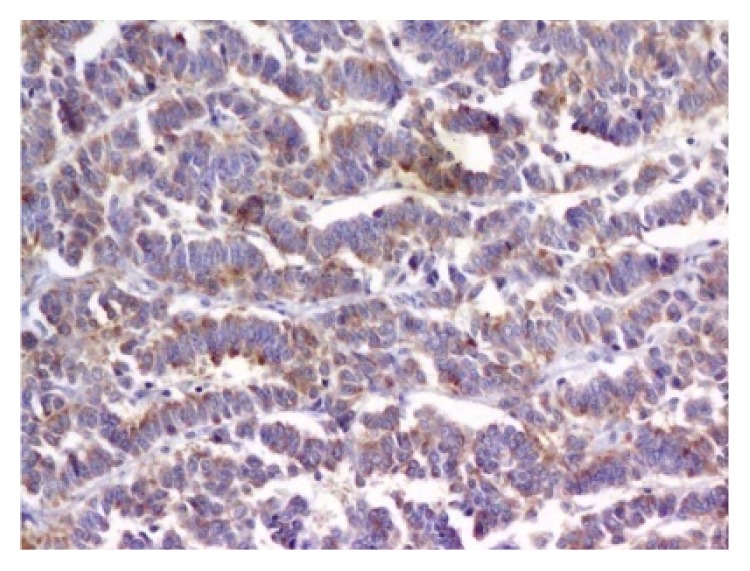
Tumor cells showing positivity for synaptophysin (immunohistochemistry for synaptophysin, ×200).

**Figure 7:**
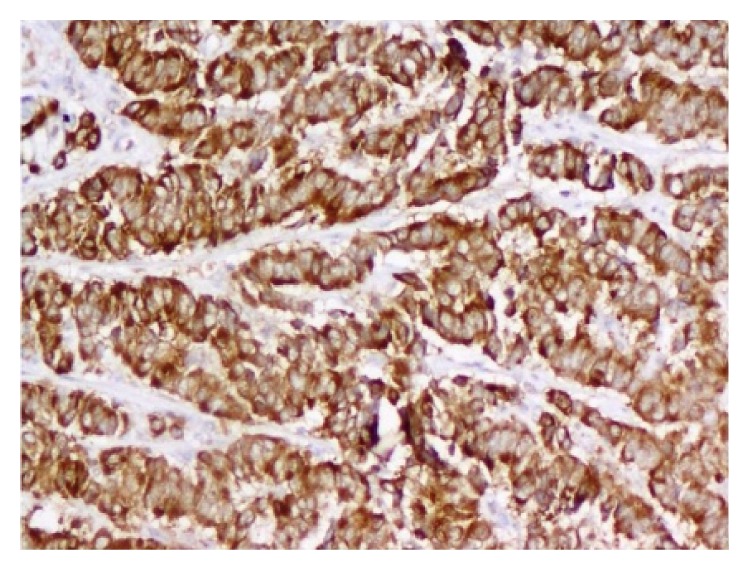
Tumor cells showing positivity for chromogranin (immunohistochemistry for chromogranin, ×200).

Rectal and ovarian biopsies were consistent with neuroendocrine carcinoma (Figures 4 and 5). The histopathology showed poorly differentiated cells with mitotic index of 42 per 10 high power fields (HPF). Immunocytochemistry for synaptophysin and chromogranin was positive (Figures 6 and 7) and also immunolabelled as Ki67 antigen positive. The Ki67 index was 76%. Hence, we reached a diagnosis of primary neuroendocrine carcinoma (NEC) of rectum or nonfunctional high grade NEC (NF HG-NEC) of rectum with distant metastasis to ovaries. As there was ovarian and liver metastasis, she had stage IV disease as per the American Joint Committee on Cancer (AJCC) staging for colorectal cancer.

The nature and stage of the disease as well as poor prognosis were explained to the patient and her relatives. She received 3 courses of palliative chemotherapy (etoposide and cisplatin) and pegfilgrastim and underwent paracentesis twice in view of abdominal distension. She subsequently developed pain in right lower limb. Doppler of right lower limb was suggestive of subacute thrombosis of bifurcation of common femoral and entire superficial and popliteal veins. Patient received enoxaparin and warfarin and she recovered. After adjusting the INR, she continued warfarin. She had regular follow-ups with medical oncologists and us, the gynecologists, for one year and has had no evidence of new growths 6 months following treatment.

## 3. Discussion

First described by Otto Lurbarsch in 1867, the neuroendocrine neoplasms are very rare with incidence around 2.5–5 per 100,000 and prevalence of 35 per 100,000. The diagnosis and monitoring of neuroendocrine neoplasms (NENs) can be challenging [1]. The NENs may form in multiple locations throughout the body, and although they share a number of common features, the clinical presentation may vary according to site of origin, secretory potential, and histological subtype. They are usually named based on their site of origin, the usual sites being intestine, pancreas, or the lung. Neuroendocrine neoplasms may be slow growing neuroendocrine tumors (NETs) or aggressive neuroendocrine cancers (NECs) that often arise in lungs and gastrointestinal tract [1–3].

Patients may be asymptomatic or present episodically with nonspecific symptoms that can be mistaken for other more common conditions, including irritable bowel syndrome (IBS), diabetes, and asthma. Many patients may have NENs for years before they are diagnosed. In fact, the estimated time to diagnosis of certain NENs can reach 5 to 7 years.

### 3.1. Classification and Staging

Traditionally, the NENs were named after the site of origin, the most common being the gastrointestinal tract (GIT) and the pancreas—the gastroenteropancreatic NENs (GEP-NENs). These are again divided into the foregut, midgut, and hindgut NENs based on the embryological development of the GIT and the arterial supply [2]. Our case study pertains to hindgut NEN, specifically rectal high grade neuroendocrine carcinoma (HG-NEC).

Several organizations like the World Health Organization (WHO), the European Neuroendocrine Tumor Society (ENETS), the American Joint Committee on Cancer (AJCC), and the North American Neuroendocrine Tumor Society (NANETS) have proposed guidelines to arrive at a more specific classification system to accurately assess prognosis of these tumors [2–8].

The World Health Organization (WHO) and the European Neuroendocrine Tumor Society (ENETS) both incorporate mitotic count and Ki-67 proliferation for the classification of gastroenteropancreatic NENs (GEP-NETs). For NENs of the lungs and thymus, the WHO includes mitotic count and assessment of necrosis [2–4].

As shown in Table 1, NENs may be classified based on their histopathology, cell differentiation, mitotic index, and immunohistochemistry into mainly two groups [2–8]:

- Well-differentiated neuroendocrine tumor (WD-NET): slow growing, mostly benign—staged by AJCC/ENETS systems.
- High grade neuroendocrine carcinoma (HG-NEC): poorly differentiated, invasive and highly metastatic—usually staged by AJCC.

Neuroendocrine neoplasms may also be functional (F) or nonfunctional (NF). Functional tumors produce hormones, which exert various clinical manifestations and include insulinomas, gastrinomas, and glucagonomas. Nonfunctional NENs cause symptoms like pain abdomen or bloating related to tumor or due to the presence of metastases [1]. These tumors are aggressive, and even when the primary tumor is small, there can be extensive locoregional spread and bulky metastases [1, 8].

### 3.2. Neuroendocrine Cancers (NECs)

Poorly differentiated (PD) or high grade (HG) neuroendocrine carcinomas (NECs) encompass many NENs including small-cell carcinoma of the lung, large-cell high grade NEC of the lung, extra pulmonary small-cell/large-cell NEC, and high grade NEC with mixed features [9, 10].

Extra pulmonary HG-NECs are very rare and elusive. They may originate anywhere in the GI tract, bladder cervix, or prostate. The prognosis is poor, with median survival periods in patients with localized and distant disease of 34 and 5 months, respectively [11, 12]. The tumor cell type (i.e., small-cell versus large-cell versus mixed) does not predict survival or prognosis.

Hindgut NECs are aggressive tumors with more than 60% having metastatic disease at the time of diagnosis. The median survival interval in affected patients ranges between 5 and 14 months [12]. Rectal HG-NECs comprise less than 1% of colorectal cancers [3].

### 3.3. Diagnosis

Radio imaging by CT/MRI scan and directed biopsy followed by histopathologic examination and immunohistochemistry help in diagnosing the disease. Endoscopy and directed biopsy play an important role in identifying smaller lesions that are sometimes asymptomatic. Unlike WD-NETs, the HG-NECs have poor somatostatin receptor expression rendering somatostatin receptor scintigraphy useless in assessing NEC. 18F-fluorodeoxyglucose positron emission tomography appears to be the best method of evaluating disease spread and guiding further treatment [13].

NEN can be an incidental finding on routine endoscopy. NENs secrete several biomarkers like chromogranin A, 5-hydroxyindoleacetic acid, neuron specific enolase, and synaptophysin. Chromogranin A is a marker that is elevated in up to 90% of patients with NENs. They have prognostic significance and can be used to help monitor disease progression and to correlate with disease burden and survival. Another marker, synaptophysin, is a membrane component of presynaptic vesicles, found in neurons and neuroendocrine cells. It also acts as a reliable marker for NENs when combined with chromogranin A [14, 15].

The high grade NECs are characterized by a high mitotic rate (more than 20 mitoses per 10 high power fields) and extensive necrosis. In fact, most of the cancers in this family have more mitoses than these values often ranging as high as 40 to 70 mitoses per 10 high power fields. The Ki-67 proliferation index is high in gastrointestinal HG-NEC (more than 20% by definition and usually 50–90%). Sometimes HG-NECs may contain elements of adenocarcinomas or squamous cells. When these components constitute more than 30% of the tumor, we refer to these as combined NECs [8–11].

The biopsies from our patient revealed poorly differentiated cells with mitotic index of 42/10HPF and Ki67 index of 76%, as well as chromogranin and synaptophysin positivity. This led to the diagnosis of high grade neuroendocrine carcinoma, HG-NEC, of rectum, an aggressive NEN that had metastasized to ovaries and liver. The primary site was determined to be the rectum as primary ovarian NEC is usually unilateral. Thus, our patient had stage IV GEP-HG-NEC (gastroenteropancreatic high grade neuroendocrine carcinoma) of rectum. Her disease staging was as per the AJCC staging of colorectal cancer.

### 3.4. Treatment Modalities for Hindgut NEC

National Comprehensive Cancer Network (NCCN), North American Neuroendocrine Tumor Society (NANETS), and European Neuroendocrine Tumor Society (ENETS) have all proposed management guidelines, whose principles are similar.

### 3.5. Locoregional Disease Management: Multimodality Approach

The HG-NECs have high proclivity to metastasize even when they are localized, and published research evidence shows that surgery alone is rarely curative [16, 17]. 

*(i) Definitive Chemoradiation Treatment [9]*. 4–6 cycles of cisplatin/carboplatin and etoposide chemotherapy and radiation is effective in locoregional disease. 

*(ii) Surgery with Adjuvant Chemotherapy/Radiation [9]*. The recommended regimen is optimal resection of locoregional disease followed by 4–6 cycles of cisplatin/carboplatin and etoposide chemotherapy.

Where the risk of local disease recurrence is higher, the recommended treatment is sequential radiation.

### 3.6. Metastatic Disease (Stage IV)/Inoperable Cases

More than half of the patients with HG-NEC have advanced metastatic disease at the time of diagnosis. The standard first-line salvage treatment for widespread poorly differentiated neuroendocrine tumors is the combination of cisplatin and etoposide [9, 18]. Palliative chemotherapy with cisplatin and etoposide for metastatic neuroendocrine carcinoma improves the survival duration [18, 19]. The general recommendation is to give 3-4 cycles of salvage chemotherapy [9]. Carboplatin based chemotherapeutic regimens have been proven successful in lung NECS, so it is assumed that they may be useful in advanced hindgut GEP HG-NECs also. Topotecan, paclitaxel, docetaxel, vinorelbine, and gemcitabine are some options for second-line management in relapsed HG-NEC [9].

Our patient had stage IV metastatic extra pulmonary HG-NEC and received 3 cycles of palliative chemotherapy with cisplatin and etoposide. The subsequent evaluation revealed complete regression of lesions and she was doing well 6 months after treatment.

## 4. Conclusion

High grade neuroendocrine carcinomas (HG-NECs) of rectum are very rare cancers that comprise less than 1% of colorectal cancers. More than half of these patients have metastatic disease at the time of diagnosis and may present with large ovarian Krukenberg tumor. Thus, they form a very rare differential diagnosis for secondary ovarian malignancy. Histopathology (high mitotic rate, presence of necrosis) and immunohistochemical markers like chromogranin, synaptophysin, and high Ki67 index help clinch the diagnosis. Palliative cisplatin-etoposide chemotherapy is the treatment advised in such advanced cases while surgical resection and chemoradiation is the current recommendation in locoregional disease.

## Figures and Tables

**Table 1:** Compiled classification and nomenclatures of NENs.

Traditional nomenclature based on grades of differentiation and histopathology		WHO; ENETs classification of NENs			Accepted nomenclature
		Types	Mitotic index per 10 high power fields (HPF)	Immunohistochemistry Ki 67 index	
Low	Carcinoid tumor	Neuroendocrine tumor (NET), grade 1	<2	<3%	Well-differentiated neuroendocrine tumor (WD-NET) May be functional (F) or nonfunctional (NF)
Intermediate	Atypical carcinoid tumor	Neuroendocrine tumor (NET), grade 2	2–20	3–20%	

High	Small-cell carcinoma Large-cell neuroendocrine carcinoma (NEC)	NEC grade 3, small-cell carcinoma NEC grade 3, large cell neuroendocrine carcinoma	>20	>20%	High grade neuroendocrine carcinoma (HG-NEC) (F or NF)
